# The Association of Serum Profile of Transferrin Isoforms with COVID-19 Disease Severity

**DOI:** 10.3390/jcm13082446

**Published:** 2024-04-22

**Authors:** Lech Chrostek, Kacper Gan, Marcin Kazberuk, Michal Kralisz, Katarzyna Janicka, Ewa Gruszewska, Anatol Panasiuk, Bogdan Cylwik

**Affiliations:** 1 Department of Biochemical Diagnostics, Medical University of Bialystok, 15-269 Bialystok, Poland; 2 Department of Gastroenterology, Hepatology and Internal Diseases, Voivodeship Hospital in Bialystok, 15-278 Bialystok, Polandanatol@panasiuk.pl (A.P.); 3 Department of Pediatric Laboratory Diagnostics, Medical University of Bialystok, 15-274 Bialystok, Poland; 4 Department of Clinical Medicine, Medical University of Bialystok, 15-254 Bialystok, Poland

**Keywords:** COVID-19, transferrin isoforms, disease severity, oxygen therapy, cytokine storm, comorbidities, prognostic marker

## Abstract

**Background/Objective**: Bearing in mind the relationship of transferrin (TRF) microheterogeneity with the biological activity of its isoforms, we propose, in this study, to determine the association of the profile of TRF isoforms with COVID-19 disease severity and to compare this profile to the profiles of other diseases. **Methods:** The disease group consisted of 96 patients from whom blood was collected twice, upon admission to the ward and after treatment (on average on the ninth day). TRF isoforms were separated by capillary electrophoresis. The analysis included disease severity, cytokine storm, comorbidities, patient survival, oxygen therapy, and modified early warning scores (MEWSs). **Results:** The concentration of 5-sialoTRF was higher in patients compared to controls at the beginning and during COVID-19 treatment. The concentration of this isoform varies with the severity of disease and was higher in critical patients than those with a moderate condition. Additionally, the level of 5-sialoTRF was lower and the level of 4-sialoTRF was higher in patients with comorbidities than that in patients without them. The concentration of 5-sialoTRF was lower and the concentration of 4-sialoTRF was higher in surviving patients than in non-surviving patients. There were no statistical changes in TRF isoforms according to presence of cytokine storm, MEWS, and oxygen therapy. **Conclusions:** We conclude that the profile of TRF isoforms in COVID-19 patients differs from that in other diseases. An increase in the concentration of a sialic acid-rich isoform, 5-sialoTRF, may be a compensatory mechanism, the goal of which is to increase oxygen delivery to tissues and is dependent on the severity of the disease. Additionally, the concentration of 5-sialoTRF may be a prognostic marker of the survival of COVID-19 patients.

## 1. Introduction

The SARS-CoV-2 virus causes a viral pneumonia that varies in severity, from mild to severe, and may cause lung damage [1]. Its dynamic spread throughout the world is mainly due to its ability to mutate [2]. One of the many clinical manifestations of COVID-19 is an acute respiratory distress syndrome (ARDS), which is a rapidly evolving condition where the body’s oxygen supply suddenly deteriorates [3,4,5]. Hemoglobin is responsible for supplying the right amount of oxygen to the tissues. Iron necessary for hemoglobin synthesis in the bone marrow is provided by transferrin (TRF). TRF is a glycoprotein synthesized in the liver that is built from a single polypeptide chain to which two complex glycans are attached by an N-glycosidic bond [6]. Depending on the structure of the glycans, different TRF isoforms are distinguished [7,8]. Glycans often have conformational variability, which may affect protein function and also modulates their interaction with receptors [9,10]. In the peripheral blood serum of a healthy population, there are five fractions of transferrin, asialotransferrin, disialotransferrin, trisialotransferrin, tetrasialotransferrin, and pentasialotransferrin, with a predominance of tetrasialotransferrin [11]. It has been documented that TRF isoforms containing more branched glycans (more sialylated) have increased affinity to the transferrin receptor during pregnancy [12,13]. Pregnancy is a physiological state requiring an increased oxygen supply to the body’s (mother and fetus) tissues and cells. Thus, the changes in glycosylation can modulate the biological activity of transferrin and, as a result, iron metabolism and oxygen delivery to the tissues.

Transferrin is an acute phase reactant [14], and the acute phase reactions are of great importance in the diagnosis and treatment of COVID-19 [15]. On the other hand, patients with COVID-19, due to respiratory failure (dyspnea), have increased requirements for oxygen. Based on this information, and the importance of transferrin isoforms in delivering oxygen to the tissues through its involvement in iron metabolism [12], the aim of this study was to evaluate the association of the serum profile of transferrin isoforms with the severity of COVID-19 disease (i.e., COVID-19 severity scale of World Health Organization, presence of cytokine storm, oxygen therapy and chronic disease, the value of modifited early worning score—MEWS) and to compare this profile to the profiles in other diseases. Based on our previous research, we have grounds to assume that the profile of TRF isoforms in COVID-19 disease will differ from profiles in other clinical situations [16,17,18,19], which creates the possibility of using this study for clinical (diagnostic) purposes.

## 2. Materials and Methods

### 2.1. Study Design

A total of 96 patients with COVID-19 participated in the study (56 men in median age 62 years; range: 22–85, 40 women in median age 64 years; range: 23–89), and they were admitted to the Department of Gastroenterology, Hepatology and Internal Diseases with the Center for Diagnostics and Endoscopic Treatment between 20 December 2021 and 16 March 2022. Initial diagnosis of COVID-19 disease was made in the hospital emergency room by the qualitative detection of SARS-CoV-2 antigen (Ag) in human-nasal-swab specimens with Panbio^PM^ COVID-19 Ag Rapid Test Device (Abbott Rapid Diagnostics Jena GmbH, Jena, Germany). Then, the infection (SARS-CoV-2-RNA) was confirmed by qualitative real-time reverse transcription polymerase chain reaction (RT-PCR) using SARS-CoV-2 Triplex PCR kita from Astra Biotech GmbH (Berlin, Germany) with thermocycler Azure Cielo 6 (Azure Biosystems, Dublin, OH, USA). After the diagnosis, the first blood collection was performed (Sample 1). The second blood collection was made after 9 days on average in the hospital ward (Sample 2). Biochemical, hematology, and coagulometric tests were performed on both blood collections, as were blood-gas and co-oximetry tests (Table 1). Transferrin isoforms also performed in both of these samples. All sick subjects signed informed consent to participate in the studies.

### 2.2. Patients Quantification

Patients were classified according to disease severity (moderate, severe and critical), Modified Early Warning Score (MEWS), the presence of cytokine storm, the presence of concomitant diseases, and the amount of oxygen therapy required.

A moderate condition of disease was defined as the presence of a fever, cough, anosmia and lack of dyspnea, as well as lower respiratory tract involvement, with oxygen saturation being measured by pulse oximetry ≥ 94% and minor radiographic changes.

A severe condition was recognized when oxygen saturation decreased below 94% at a respiratory rate below 30/min, while radiologically pulmonary infiltrates increased above 50% with respiratory failure.

The condition was diagnosed as critical when multi-organ dysfunction with respiratory failure occurred (according to WHO, 2020) [20].

The moderate severity of the disease was diagnosed in 63 patients, and it was severe in 14 patients, severe/critical in 2 patients, and critical in 17 patients. Additionally, 13 of 96 patients did not survive (mortality—13.5%), while 9 were transferred to the intensive care unit (9.4%).

Cytokine storm, defined as having interleukin-6 (IL-6) levels above 100 μg/mL, was diagnosed in 26 patients. A total of 66 patients had concomitant disease, among which the following predominated: hypertension—in 21 patients, hypertension and type 2 diabetes mellitus—in 10, cirrhosis—in 8 and diabetes mellitus—in 5 patients, and others. The relationship between the severity of the disease and the presence of comorbidities is as follows: chronic diseases were present in 39 patients in moderate condition, in 11 patients in severe condition, and in 16 patients in critical condition.

A Modified Early Warning Score (MEWS) was calculated on the basis of five parameters, including systolic blood pressure, heart rate, respiratory rate, temperature, and the alert, verbal, pain, unresponsive neurological score (AVPU) [21]. Each of the MEWS components were ranked on a scale from 0 to 3 points based on the degree of derangement of the parameter. The total score was the sum of each component.

A total of 76 patients used oxygen therapies, with low-flow nasal cannula oxygen (up to 18 L/min at an oxygen concentration of 25–35%) being utilized in 43 patients, high-flow nasal cannula oxygen (oxygen flow 20–60 L/min, concentration set depending on oxygen saturation 40–100%) in 23 patients, and respiratory therapy in 10 patients. Of the 63 patients in a moderate condition, the majority—37 patients—had a low-oxygen flow, while 20 patients did not require oxygen therapy, and 6 patients had a high-oxygen flow. However, most of the patients (11) in a severe condition were connected to a high-oxygen flow, and only 3 patients were connected to a low-oxygen flow. In turn, 10 patients in critical condition were connected to a respirator, 5 had a high-oxygen flow, and 2 patients had a low-oxygen flow.

The control group consisted of 30 healthy subjects (15 men and 15 women) aged 21–65. The study was in accordance with the Helsinki Declaration and was approved by the Bioethical Committee at the Medical University of Bialystok (Approval No. APK.002.583.2021).

### 2.3. Analysis

Biochemical tests (creatinine, creatinine kinase—CK, alaninę aminotransferase—ALT, aspartate aminotransferase—AST, γ-glutamyltransferase—GGT, lactate dehydrogenase—LDH, bilirubin, glucose, cholesterol and triglycerides—TG) were performed on a Cobas 6000 analyzer and a C 501 module (Hitachi, Tokyo, Japan), while hematology tests were conducted on an XN-1000 analyzer (Sysmex Corporation, Singapore), coagulometric tests (INR, fibrinogen and prothrombin time—PT) on an ACL TOP 300 CTS analyzer (Instrumentation Laboratory, Werfen Company, Bedford, MA, USA), and blood gas and co-oximetry on an ABL 90 FLEX PLUS analyzer (Radiometer Medical ApS, Brønshøj, Denmark). IL-6 was determined by electrochemiluminescence assay on COBAS e-411(Roche Diagnostics International Ltd., Rotkreuz, Switzerland).

### 2.4. Transferrin Isoforms Determination

Determination of TRF isoforms was performed by the capillary electrophoresis (CE) on a MINICAP electrophoretic system using a MINICAP CDT reagent kit (Sebia, Evry, France). With this technique, charged molecules are separated by their electrophoretic mobility in an alkaline buffer with a specific pH 8.8. The human-serum TRF isoforms are detected as five major fractions in the following order: asialotransferrin (a-sialoTRF), disialotransferrin (2-sialoTRF), trisialotransferrin (3-sialoTRF), tetrasialotransferrin (4-sialoTRF), and pentasialotransferrin (5-sialoTRF).

### 2.5. Statistics

Results were expressed as median and ranges. The Shapiro–Wilk test was used to assess the distribution of data. For tests such as haemoglobin, RBC, MCV, PLT, pO_2_ and RR diastolic, the comparisons with the control group were performed by a t-Student test (due to their normal distribution as assesed by Kolmogorov-Smirnov test). The non-parametric Mann−Whitney U test was used for the rest of the tests. The differences between patients (Samples 1 and 2) were evaluated by a paired *t*-test. The effect of the disease severity, oxygen therapy, and modifited early worning score (MEWS) on the concentration of TRF isoforms was tested by the ANOVA rank Kruskal–Wallis test. The Spearman’s rank correlation coefficient was used to assess the correlation between transferrin isoforms and laboratory tests. We considered *p* values < 0.05 to be statistically significant.

## 3. Results

### 3.1. Transferrin Isoforms between Samples

**Figure 1:**
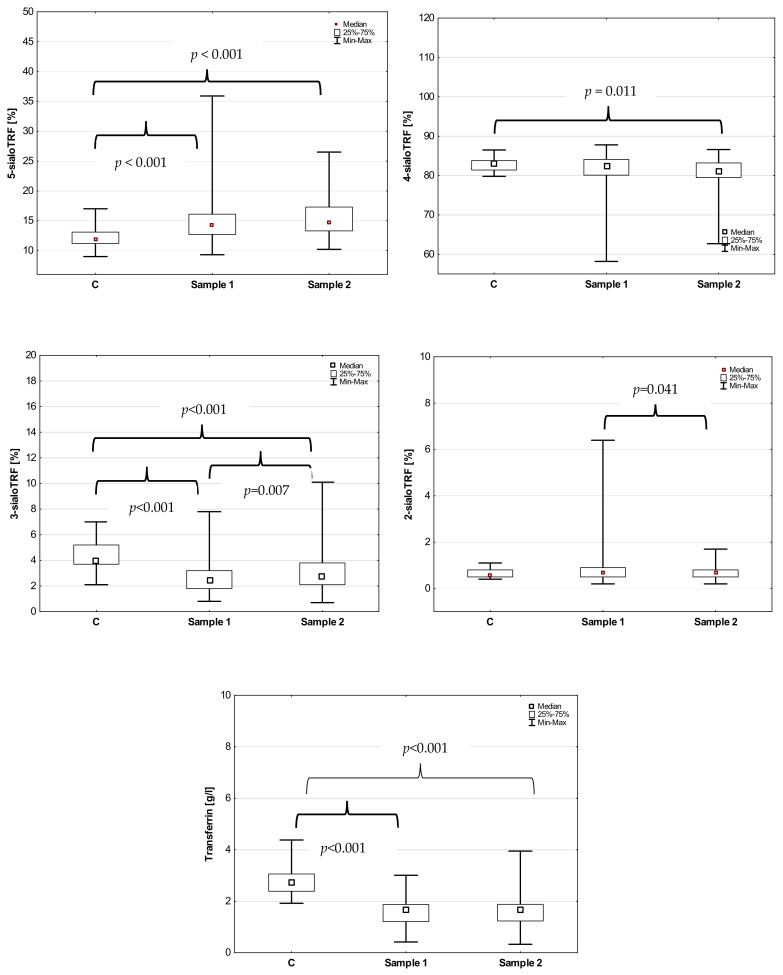
Transferrin isoforms and total transferrin concentration in the control group (C) and COVID-19 patients at the beginning of the treatment (sample 1) and after the treatment (sample 2).

Among transferrin isoforms, the concentration of 5-sialoTRF was significantly higher (14.2%; range: 9.3–35.9) and the concentration of 3-sialoTRF significantly lower (2.4%; range: 0.8–7.8) in Sample 1 than that in the controls (11.95%; range: 9–17 for 5-sialoTRF and 4.0%; range 2.1–7.0 for 3-sialoTRF) (*p* < 0.001 for both comparisons). In Sample 2, there were statistically significant increases in the 5-sialoTRF concentrations (14.7%; range: 10.2–26.4) and significant decreases in 4-sialoTRF (81.15%; range: 62.7–86.6) and 3-sialoTRF (2.7%; range: 0.7–10.1) in comparison with the control group (*p* < 0.001, *p* = 0.011 and *p* < 0.001, respectively) (Figure 1). While comparing the concentrations of transferrin isoforms between the samples, there was a significant decrease in the concentration of 3-sialoTRF and a significant increase in the concentration of 2-sialoTRF in Sample 2 in comparison to Sample 1 (*p* = 0.007 and *p* = 0.040, respectively).

### 3.2. Total Transferrin between Samples

Total transferrin concentrations were significantly lower at the beginning of the study (Sample 1) (1.66 g/L; range: 0.42–3.01) and after the treatment (Sample 2) (1.67 g/L; range: 0.33–3.95) in comparison with the controls (*p* < 0.001 for both comparisons) (Figure 1). There was no significant difference in transferrin levels between the samples (*p* = 0.690).

### 3.3. Transferrin Isoforms According to the Severity of Disease

Among transferrin isoforms, only 5-sialoTRF changed with the severity of disease (H = 10.228; *p* = 0.017), being significantly higher in critical (16.75%; range: 12.10–35.90) patients than those with a moderate condition (14.10%; range:9.30–26.50) (*p* = 0.009). These changes were similar to the total transferrin concentration. In critical conditions, the transferrin level (1.6 g/L; range: 0.42–1.73) was significantly lower than that in moderate conditions (1.75 g/L; range: 0.68–2.90) (*p* < 0.001).

Although the total transferrin concentration decreases in patients with cytokine storm (median in cytokine storm—1.2 g/L, median in patients without cytokine storm—1.7 g/L) (*p* < 0.001), we did not observe changes in the concentrations of any of the transferrin isoforms (*p* = 0.182, *p* = 0.365, *p* = 0.659 and *p* = 0.754 for 5-sialoTRF, 4-sialoTRF, 3-sialoTRF and 2-sialoTRF, respectively). However, there were statistically significant correlations between IL-6 and transferrin isoforms with 5-sialoTRF (R = 0.289, *p* = 0.005) and with 4-sialoTRF (R = −0.252, *p* = 0.016). IL-6 also correlated with the total concentration of transferrin (R = −0.500, *p* < 0.001).

The presence of chronic disease affected the level of 5-sialoTRF and 4-sialoTRF but not the total concentration of transferrin. The level of 5-sialoTRF was significantly lower (13.8 vs. 14.95%) (*p* = 0.042) and the concentration of 4-sialoTRF significantly higher (83.1 vs. 81.6%) (*p* = 0.012) in patients with concomitant chronic diseases than that in patients without them.

**Figure 2:**
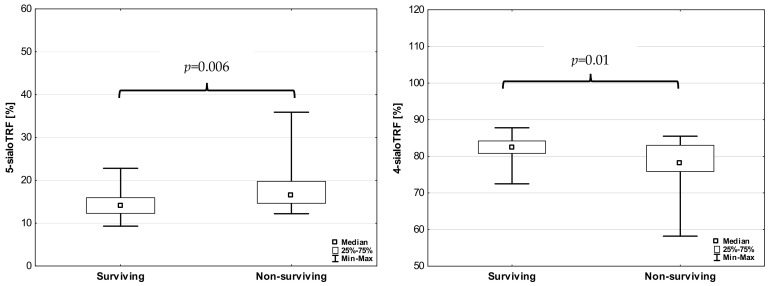
Transferrin isoforms in surviving and non-surviving COVID-19 patients.

None of transferrin isoforms changed in COVID-19 patients depending on the modifited early warning score (MEWS) (*p* = 0.327, *p* = 0.110, *p* = 0.064 and *p* = 0.544, respectively, for 5-sialoTRF, 4-sialoTRF, 3-sialoTRF and 2-sialoTRF). However, the results of 5-sialoTRF and 4-sialoTRF in surviving patients were dependent on their results in the second sample (post-treatment) (Figure 2). The level of 5-sialoTRF was significantly lower and the level of 4-sialoTRF was significantly higher in surviving patients than in non-surviving patients (*p* = 0.010 and *p* = 0.006, respectively).

Taking oxygen therapy into account, only the total transferrin concentration statistically significantly differed depending on the applied oxygen/respiratory therapy, being significantly lower in patients with a high-oxygen flow (1.37 g/L, range: 0.56–3.01) and undergoing respiratory therapy (1.36 g/L; range: 0.50–1.73) than in patients without oxygen therapy (1.91 g/L; range: 0.94–2.79) (*p* = 0.037 and *p* = 0.020, respectively).

## 4. Discussion

Infection with SARS-CoV-2 stimulates the liver to synthesis acute-phase reaction proteins (APRPs) [3]. Many of these are positive proteins of the acute-phase, but among these are also transferrin and albumin, which are negative acute-phase proteins [22]. As expected, the total concentration of transferrin diminished in patients with COVID-19 at the onset of the treatment (Sample 1) and remained reduced after treatment (sample 2). There was no statistically significant difference between the two timepoints collected during treatment. The persistent changes in the total concentration of transferrin can be explained by the half-life of transferrin in the blood, which is 14 days and is longer than the time interval between the 1st and 2nd collection of the material for the study (an average of 9 days). Our previous research indicates that the altered serum profile of transferrin isoforms may be pathognomonic for a particular disease [16,17,18,19], and so, in patients with chronic hepatitis, the tetrasialotransferrin level was increased and the pentasialotransferrin level decreased [16]. Additionally, in rheumatoid arthiritis patients, there was a significant decrease in the relative concentrations of trisialo- and pentasialotransferrin and a significant increase in tetrasialotransferrin [17]. Meanwhile, in primary biliary cholangitis there was a lower concentration of disialotransferrin and trisialotransferrin [18] and a significant decrease in the concentration of pentasialotransferrin in both acute and chronic pancreatitis, as well as a significant increase in tetrasialotransferrin in the acute pancreatitis group in comparison to the healthy subjects [19]. In this study, a picture of transferrin isoforms emerged featuring an increase in 5-sialoTRF and a decrease in 3-sialoTRF. These changes persisted during treatment. Thus, in COVID-19 patients, the transferrin isoform profiles differed compared to other clinical situations, which may be used for diagnostic purpose. Additionally, we noted a decrease in the concentration of the largest fraction of transferrin, 4-sialoTRF, after the treatment compared to controls. An elevated level of 5-sialoTRF depends on the severity of the disease being higher in critically ill patients than that in moderately ill patients. This change is reflected in the total concentration of transferrin, which is lower in critical states than in the moderate states. Interestingly, the survival of patients was associated with the concentration of 5-sialoTRF measured after the treatment. In surviving patients, the level of 5-sialoTRF was lower than that in non-surviving patients. These results may be promising in regard to recognizing 5-sialoTRF, alongside other acute-phase proteins such as procalcitonin, CRP and ferritin, as a prognostic marker of COVID-19 patients’ severity and survival rates [23,24,25]. The previously mentioned acute-phase proteins were higher in severe cases of COVID-19 and had an impact on the survival of patients with COVID-19.

Patients with ARDS need oxygen therapy [26], and an important link in the supply of oxygen to tissues is transferrin. As mentioned in the results, in the course of COVID-19 disease, the total concentration of transferrin is lower than that in the milder course. This is undoubtedly related to higher inflammation in more severe disease, and thus patients with low transferrin concentrations require a greater supply of oxygen in the form of oxygen therapy than patients with higher transferrin concentrations. The total transferrin concentration was lower in patients with a high-oxygen flow and undergoing respiratory therapy than in patients with low-oxygen therapy. Increasing the share of the highly sialylated isoform of transferrin (5-sialoTRF) seems to be a compensatory mechanism aimed at reducing tissue hypoxia in the severe form of the disease.

The presence of comorbidities interferes with the underlying disease (worsening the conditions of patients) and affects patient outcomes. Since most of these diseases do not seem to affect the liver as a site of transferrin synthesis, no statistically significant difference in total transferrin concentration between patients with and without chronic diseases was found. Surprisingly, it turned out that comorbidities affect the levels of transferrin isoforms. A lower concentration of 5-sialoTRF in patients with an accompanying chronic disease should therefore be treated as an additional burdening factor for patients with COVID-19, as these isoforms have a lower affinity for transferrin receptors, which intensifies respiratory disorders in these patients [11,12].

None of transferrin isoforms showed an association with the modified early warning scale (MEWS), which is a widely used diagnostic tool for identifying patients in life threatening states [21]. However, the fact is that neither heart rate (HR), saturation (Sat0_2_), or diuresis (eGFR) correlated with the concentrations of transferrin isoforms or with the total concentration of transferrin. The only exception was the negative correlation of 5-sialoTRF concentration with body temperature (R = −0.248, *p* = 0.018).

Another surprise was that none of the transferrin isoforms concentration changed depending on the presence of the cytokine storm, although the concentration of total transferrin decreased in the conditions of the cytokine storm. Correlation analysis provided information about negative correlation IL-6 with total concentration of transferrin but also about positive correlation of IL-6 with the level of 5-sialoTRF and negative correlation of IL-6 with 4-sialoTRF. It is understandable that the increasing level of IL-6 intensifies the acute-phase reaction, which is associated with a decrease in the concentration of the negative acute-phase protein, which is a transferrin (negative correlation). This is also consistent with the fact that the concentration of 5-sialoTRF increases during the course of the disease. Therefore, IL-6 positively correlates with 5-sialoTRF. Although the level of 4-sialoTRF did not decrease significantly at the beginning of the treatment (sample 1), it showed a downward trend, which was already shown in the negative correlation with IL-6.

Transferrin, by providing iron for hemoglobin synthesis, plays a key role in supplying tissues with oxygen. This process becomes particularly important in conditions involving increased demand for oxygen, which is characteristic for patients with acute respiratory failure. Finally, in these patients, a decreased hemoglobin concentration is to be expected; such a change was demonstrated in this study. Since the half-life of hemoglobin coincides with the lifetime of erythrocytes, its lowered concentrations in both samples did not differ from each other.

A limitation of the study is the small size of the group, which does not allow categorization of patients according to various comorbidities that influence the concentration of transferrin isoforms.

## 5. Conclusions

Given the importance of transferrin in the delivery of oxygen to tissues, an increase in the concentration of the sialic acid-rich isoform, 5-sialoTRF, is a compensatory mechanism, the goal of which is to increase hemoglobin synthesis and, as a result, increase oxygen delivery to tissues. An additional premise regarding the importance of 5-sialoTRF is the dependence of its concentration on the severity of the disease, which can promote this transferrin fraction as a prognostic marker of COVID-19 patients’ survival. The differences in the transferrin isoforms in COVID-19 patients compared to other diseases can be used diagnostically.

## Figures and Tables

**Table 1:** Characteristics of tested group.

	Control Group		Sample 1		Sample 2	
Test	Median	IQR	Median	IQR	Median	IQR
pH	7.405	0.50	7.457 *	0.058	7.459 *	0.052
pO_2_ [mmHg]	97.0	8.0	83.6 *	43.0	82.6 *	38.1
pCO_2_ [mmHg]	39.0	4.0	36.1 *	5.8	38.5	6.4
SatO_2_ [%]	97.0	1.0	96.8	5.8	96.7 *	3.2
BE [mEq/L]	0.33	2.05	2.50 *	5.60	3.00 *#	4.10
IL6 [pg/mL]	1.5	0.32	42.0 *	107.6	24.6 *	53.1
Fibrinogen [mg/dL]	294.0	86.0	393.0 *	351.0	315.5	1714
Creatinin [mg/dL]	0.86	0.18	0.85	0.53	0.77 *#	0.35
CK [IU/L]	89.0	71.0	93.0 *	145.0	32.0 *#	43.0
ALT [IU/L]	10.20	68.0	30.75 *	40.75	35.00 *	36.0
AST [IU/L]	20.0	6.0	42.0 *	47.25	33.3 *#	30.2
GGT [IU/L]	14.5	10.0	53.5 *	87.5	75.0 *	119.0
LDH [IU/L]	155.0	42.0	268.5 *	195.0	182.0 *#	92.0
Bilirubin [mg/dL]	0.46	0.23	0.52	0.38	0.447	0.626
Glucose [mg/dL]	86.4	11.6	109.0 *	42.0	105.5 *	39.3
HBG [g/dL]	13.70	1.60	12.95 *	2.80	12.60 *	2.90
HCT [%]	39.15	5.25	38.40 *	7.90	37.60 *	7.30
RBC [×10^6^/μL]	4.59	0.605	4.275 *	0.97	4.130 *	0.855
MCV [fl]	86.55	5.85	89.20	8.55	89.55 #	7.55
WBC [×10^3^/μL]	6.49	1.75	7.20	4.35	6.95 #	4.73
PLT [×10^3^/μL]	243.0	55.0	205.5	107	254.0 #	187.0
INR	0.93	0.065	1.145 *	0.29	1.18 *	0.35
PT [s]	12.40	0.70	13.75 *	3.40	14.00 *#	3.70
Cholesterol [mg/dL]	197.65	57.2	141.65 *	62.65	140.70 *	72.4
TG [mg/dL]	91.20	54.40	137.60 *	75.9	164.35 *	106.5

* Significant difference in comparison to the control group, # significant difference between the sample 1 and sample 2, IQR (interquartile range) = Q3–Q1, Q1—first quartile, Q3—thrid quartile.

## Data Availability

The raw data supporting the conclusions of this article will be made available by the authors on request.
